# Editorial: Brief research reports in experimental pharmacology and drug discovery: 2022

**DOI:** 10.3389/fphar.2023.1253015

**Published:** 2023-08-21

**Authors:** Tahsin Hassan, Aamina Yousuf, Naseer A. Kutchy

**Affiliations:** ^1^ Department of General Medicine, William Harvey Hospital, Ashford, United Kingdom; ^2^ Sher-i-Kashmir Institute of Medical Sciences and Technology, Srinagar, India; ^3^ Department of Animal Sciences, The State University of New Jersey, Rutgers, NJ, United States

**Keywords:** anthelmintics, drug, discovery, pharmacology, screening, testing

Drug discovery is the process of identifying and designing new drugs to treat diseases. The drug discovery process involves stages such as identification of the target, compound discovery, preclinical testing, and, finally, clinical testing. In this editorial, we present the summary of all the articles published on recent findings and advancements in experimental pharmacology. In these published articles, we have information about drug discovery based on experimental techniques and technologies to identify and optimize potential drug candidates. In addition, these reports cover topics such as novel drug targets, innovative drug delivery systems, drug screening methods, and pharmacological studies to understand disease mechanisms.

According to Brooks et al., angiotensin-converting enzyme 2 (ACE2) is an established receptor and entry point for both SARSCoV-1 and the novel SARS-CoV-2. The spike proteins on the viral envelope bind the ACE2 receptor, and the virus replicates efficiently in cells expressing ACE2. Given the widespread abundance of ACE2 in tissue epithelial and endothelial cells such as lungs, intestines, kidneys, and the brain, in respect to the role of ACE2 as the entry site for SARS-CoV-2, there has been much speculation regarding whether ACE inhibitors and/or angiotensin receptor blockers (ARBs) may alter ACE2 tissue abundance and thereby change the risk of transmission or development of severe complications. However, it is unclear whether ACE1 inhibitors (e.g., lisinopril) or angiotensin receptor blockers (e.g., losartan) alter tissue ACE2 expression. Brooks et al. sought to determine whether lisinopril or losartan, as monotherapies or in combination, changes tissue levels of ACE2 in healthy male and female mice. They used 40 male and 40 female mice that were 8 weeks old and treated for 21 days with drinking water containing either lisinopril, losartan, their combination, or no drug (vehicle control). On day 21, 40 animals were euthanized for collection of plasma and tissues, while the others transitioned to standard drinking water for an additional 21 days to assess whether drug-induced changes in ACE2 resolve after drug cessation. The collected specimens underwent different procedures for measurement of ACE2 protein index, measurement of Ace2 gene expression, immunohistochemistry of tissue sections, and measurement of plasma renin activity. It was interesting to see that the ACE2 protein index (which persisted 21 days after discontinuation of the drug) and the Ace2/Gapdh transcript differed significantly by tissue; it was highest in the small intestine, followed by the kidney (kidney ACE2 levels were higher in male rats than in female rats), lung, and brain. Lisinopril treatment raised the ACE2 protein index in the tissues, but the combination of lisinopril and losartan did not. In turn, lisinopril and losartan combination treatment suppressed Ace2 gene expression in the tissues.

Roquini et al. demonstrated that helminthic infections affect a huge portion of the human population worldwide, the majority being poor people living in economically susceptible places. Among these parasitic diseases is human angiostrongyliasis, caused by the nematode *Angiostrongylus cantonensis*. Roquini et al. designed a study that aimed to evaluate the antiparasitic and molecular properties of the major available anthelmintic drugs against *A. cantonensis in vitro*. It was revealed that these drugs showed a concentration- and time-dependent effect on the larvae, which was measured by a larval motility assay, currently the method of choice to evaluate the drug sensitivity of different nematode species. The group found that ivermectin caused larval immotility in the first 2 h, and when concentration was increased, arrest was almost instantaneous. Selamectin, moxidectin, and levamisole caused larval immotility in 6,12, and 2 h, respectively. However, when parasites were exposed to pyrantel pamoate or albendazole, there was a maximum loss of spontaneous movement within 2 and 24 h, respectively. It was also noted that mebendazole and fenbendazole lacked activity against *A. cantonensi*s. Their findings contribute to the existing knowledge on potential drug options and can aid in the development of targeted therapies for this parasitic disease.

The research article by D’Ercole et al. described serelaxin, a recombinant human H2 relaxin. Relaxin, a natural peptide hormone, has been found to have anti-fibrotic activity; hence, if developed successfully, analogues could be useful in catering to different pathological conditions, including cardiovascular diseases. In view of the above, the group tried to design some conformationally variable peptides over the receptor-binding domain of human H1 relaxin. Peptides, some linear and some stapled by triazole bonds, were created, and their effects were studied in cells expressing relaxin receptors. Despite the favorable premises, none of the tested H1 peptides, whether linear or stapled, revealed a substantial affinity to RXFP1 nor displayed any RLX-like biological effects. The group showed significant cAMP elevation and ERK1/2 phosphorylation in RXFP1-expressing cells; however, B7-33, a linear H2 relaxin analogue, produced effects similar to serelaxin, suggesting a difference in the B chains of H1 and H2 relaxins. In addition, their study in turn revealed that triazole conformational changes have a negative impact on H1 relaxin activity. Overall, their findings provide valuable insights into the design of relaxin agonists and highlight the importance of preserving the unique properties of H1 relaxin when developing synthetic analogues. This knowledge can guide future research and aid in the development of more effective therapeutic agents targeting the relaxin system.

In their research article, Zubkov et al. detailed the relationship between acute stressful situations to depression, anxiety disorders, and post-traumatic stress disorder (PTSD) in humans. As there are little data on possible treatment options to alleviate acute stress symptoms and prevent harmful long-term effects, developing efficacious treatment is an urgent task. The group analyzed the effects of different substances such as melatonin, neuropeptide Y (NPY), orexin, oxytocin, and clomipramine in acute stressful situations. Acute stress was induced in rats after an anhedonia test and forced swimming test. The control and test groups were selected, and the effects of different substances were observed by serum corticosteroid measurements and the sucrose preference index. It was noted that clomipramine, NPY, and oxytocin had a positive effect on acute stressful behavior as they all decreased the immobilization time in the experimental animals; however, no such response was seen with melatonin and orexin. It is very interesting that none other than a single dose of NPY decreased the levels of corticosteroids post-stress. This study further highlights the potential of intranasal NPY as a promising option for addressing specific aspects of acute stress while emphasizing the need for additional investigations to validate its effectiveness and broaden our understanding of its therapeutic applications.

Wang et al. investigated the immunomodulatory effects and underlying mechanisms of bioactive peptides derived from Xinjiang fermented camel milk. They utilized network pharmacology and molecular docking approaches to explore the potential interactions between the bioactive peptides and immune-related targets. The group showed that several bioactive peptides derived from Xinjiang fermented camel milk exhibit potential immunomodulatory effects, and network pharmacology analysis revealed specific targets and pathways involved in immune regulation. In addition, molecular docking simulations confirmed the binding interactions between the bioactive peptides and immune-related targets having the potential to modulate the immune system through direct interactions with key immune molecules. Their findings contribute to our understanding of the potential therapeutic applications of these peptides in immune-related disorders and highlight their potential as natural immunomodulators. Further experimental studies are warranted to validate these predictions and explore the clinical implications of Xinjiang fermented camel milk-derived bioactive peptides.

Saran et al. investigated the impact of mTOR (mammalian target of rapamycin) inhibitors on the function of a protein called sodium taurocholate cotransporting polypeptide (NTCP). NTCP is responsible for transporting bile acids into liver cells, and its proper functioning is crucial for maintaining liver health. The group used liver cell lines and exposed them to different mTOR inhibitors commonly used in clinical settings. They assessed the impact of these inhibitors on NTCP expression, function, and intracellular localization. The group showed that mTOR inhibitors could influence NTCP expression levels, leading to a decrease in its abundance. Furthermore, the inhibitors affected NTCP localization within the liver cells, potentially impairing its ability to transport bile acids effectively, suggesting the negative impact of mTOR inhibitors on NTCP function as therapeutic agents, particularly in liver-related conditions. Further research is needed to better understand the underlying mechanisms and assess the clinical implications of these findings.

This editorial summarizes the various articles on the advancements in experimental pharmacology and drug discovery. We believe that the findings presented in the research topic can help to advance our understanding of novel drug targets, innovative drug delivery systems, drug screening methods, pharmacological studies, and disease mechanisms. We appreciate all the contributors to these research articles, including the authors, reviewers, and the editorial team at Frontiers in Pharmacology.

